# A case of pancreatic ductal adenocarcinoma with enteroblastic, neuroendocrine, and squamous differentiation with p53 overexpression and loss of Rb expression

**DOI:** 10.1016/j.ijscr.2024.109854

**Published:** 2024-06-06

**Authors:** Takayuki Kodama, Akiho Tani, Hisoka Yamane, Tomoo Itoh

**Affiliations:** a Division of Pathology, Department of Pathology, Kobe University Graduate School of Medicine, Kobe 650-0017, Japan; b Department of Diagnostic Pathology, Kobe University Hospital, Kobe 650-0017, Japan; c Department of Hepato-Biliary-Pancreatic Surgery, Kobe University Hospital, Kobe 650-0017, Japan; d Department of Surgery, Seirei Mikatahara General Hospital, Hamamatsu 433-8558, Japan

**Keywords:** Pancreatic ductal adenocarcinoma, Hepatoid, Enteroblastic differentiation, Neuroendocrine carcinoma, Rb

## Abstract

**Introduction:**

Herein we report a case of an extremely rare pancreatic adenocarcinoma with enteroblastic differentiation (AED), an underrecognized histological subtype. Moreover, the tumor was mixed with a neuroendocrine carcinoma (NEC), which is also a rare malignancy in the pancreas.

**Case presentation:**

The patient was an elderly male who was incidentally diagnosed with a 35 mm-sized pancreatic head tumor and underwent pancreatoduodenectomy. Histopathologically, the tumor was composed of four different types: conventional ductal adenocarcinoma, AED, NEC, and squamous cell carcinoma. Interestingly, p53 overexpression and loss of Rb expression, which are characteristic findings of NEC, were observed in all components. He had been received adjuvant chemotherapy after the surgery, however, he died of bath-related cardiac arrest 14 months after surgery.

**Discussion:**

In the stomach, AED, a carcinoma resembling fetal gut epithelium, is a rare but established subtype and is considered a related entity of hepatoid carcinoma (HAC). However, gastric AED and HAC differ to some extent. In contrast to the stomach, extragastric AED, including pancreatic AED, is extremely rare, and its biological features are unclear. A mixed tumor with NEC is a complex phenomenon, but it is occasionally reported in extragastric AED. The histogenesis of mixed AED-NEC can be resolved by determining p53 and Rb status.

**Conclusion:**

Owing to their rare and novel nature, extragastric AED is under-recognized or confused with HAC. Further studies and the establishment of an extragastric AED classification are required.

## Highlights

• Pancreatic adenocarcinoma with enteroblastic differentiation (AED) is extremely rare.
• Clinicopathological features of extragastric AED, including pancreatic AED, are unclear.
• Extragastric AED is occasionally mixed with a neuroendocrine carcinoma (NEC) component.
• Combination with NEC component seems to worsen the patient prognosis.
• Rb expression can be a key that reveals mixed AED-NEC histogenesis.

## 1 Introduction

Pancreatic ductal adenocarcinoma (PDAC) is the most common adult pancreatic malignancy. Most PDAC are well-to-moderately differentiated adenocarcinomas; however, many histological subtypes, such as adenosquamous carcinoma, hepatoid carcinoma, and undifferentiated carcinoma, are listed in the 5th edition of the World Health Organization (WHO) classification of digestive system tumors [1]. Herein, we report a case of pancreatic adenocarcinoma with enteroblastic differentiation (AED), a carcinoma histologically and immunohistochemically resembling fetal gut epithelium [[1], [2], [3]], an extremely rare and under-recognized subtype in the pancreas. Moreover, our case was combined with neuroendocrine carcinoma (NEC), which is also a rare histological type in the pancreas.

Owing to its rarity, the clinical features and management of pancreatic AED are unclear. Understanding this malignancy can optimize patient care. This report was written in accordance with the SCARE criteria [4].

## 2 Case presentation

**Fig. 1:**
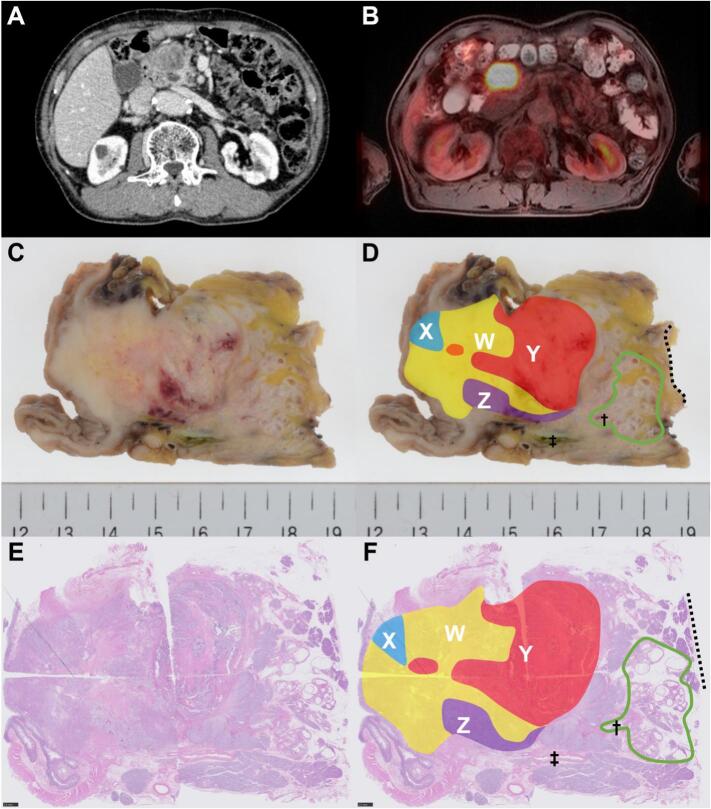
Radiological, macroscopic, and loupe-magnification images of the tumor. A, Contrast-enhanced computed tomography. A 35 mm-sized poorly enhanced mass was found in the pancreatic head. B, Positron emission tomography-magnetic resonance imaging showed high fluorodeoxyglucose accumulation (SUV 9.52). C—F, Macroscopic (C, D) and loupe-magnification images (E, F) of the resected specimen with and without annotation. A 35 mm-sized whitish solid mass was found in the pancreatic head. The mass was composed of three different histological types; pancreatic ductal adenocarcinoma (yellow, W), squamous cell carcinoma (blue, X), adenocarcinoma with enteroblastic differentiation (red, Y), and neuroendocrine carcinoma (purple, Z). Intraductal papillary mucinous neoplasm was on the distal aspect of the specimen (green circle). Black dot line, pancreatic neck margin; †main pancreatic duct; ‡common bile duct. (For interpretation of the references to colour in this figure legend, the reader is referred to the web version of this article.)

The patient was a 71-year-old Japanese male with history of atrial fibrillation and aortic regurgitation. During the evaluation of aortic regurgitation, a 35 mm-sized and poorly enhanced pancreatic head tumor was incidentally detected on computed tomography (Fig. 1A). Positron emission tomography showed high fluorodeoxyglucose accumulation (SUV 9.52, Fig. 1B). Blood tests revealed mildly elevated carcinoembryonic antigen (6.9 ng/ml); however, carbohydrate antigen 19–9, aspartate aminotransferase, alanine aminotransferase, bilirubin, and amylase levels were normal. After confirmation of carcinoma by endoscopic ultrasound-guided biopsy, open pancreatoduodenectomy was performed using the modified Child method. The operative time was 534 min, and the blood loss was 600 ml. The patient developed postoperative pancreatic fistula that was treated conservatively and was discharged 2 months postoperatively.

**Fig. 2:**
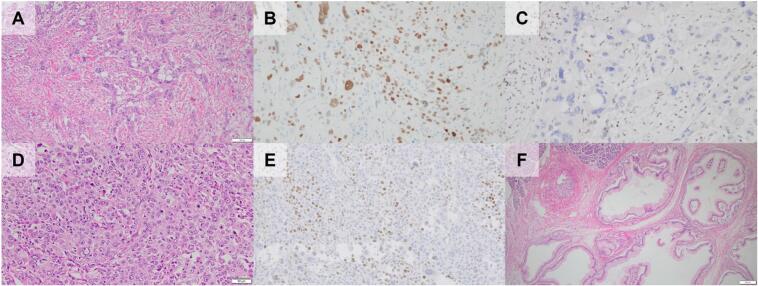
Microscopic images of the pancreatic ductal adenocarcinoma (PDAC) component/ component W (A–C), squamous cell carcinoma (SCC) component/component X (D, E), and concomitant intraductal papillary mucinous neoplasm (IPMN, F). A, hematoxylin-eosin staining of PDAC component. The lesion is composed of atypical epithelial cells with eosinophilic cytoplasm and prominent nucleoli showing an irregular trabecular structure or poorly defined glands and surrounded by desmoplastic stroma. Scale bar = 100 μm. Immunohistochemically, the component showed p53 overexpression (B) and loss of Rb expression (C). D, hematoxylin-eosin staining of the SCC component. The lesion was composed of sheet-like growth of polygonal cells with relatively well-defined cell borders. Scale bar = 50 μm. Immunohistochemically, the component showed focal p40 expression (E). p53 overexpression and loss of Rb expression were also observed (data not shown). F, hematoxylin-eosin staining of IPMN. The lesion was covered by gastric foveolar-type epithelium with low-grade dysplasia forming finger-like papillae. Scale bar = 200 μm.

**Fig. 3:**
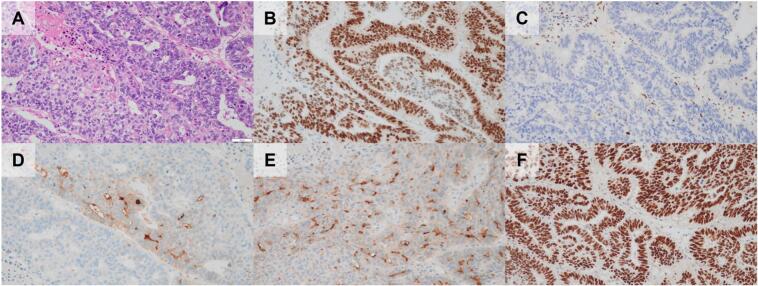
Microscopic images of the adenocarcinoma with enteroblastic differentiation component/component Y. A, hematoxylin-eosin staining. The component is composed of tubulopapillary growth of tall columnar cells with clear-to-granular cytoplasm and centrally located nuclei. Scale bar = 50 μm. Immunohistochemically, the component showed p53 overexpression (B), loss of Rb expression (C), expression of alpha-fetoprotein (D), glypican 3 (E), and spalt-like transcription factor 4 (F).

**Fig. 4:**
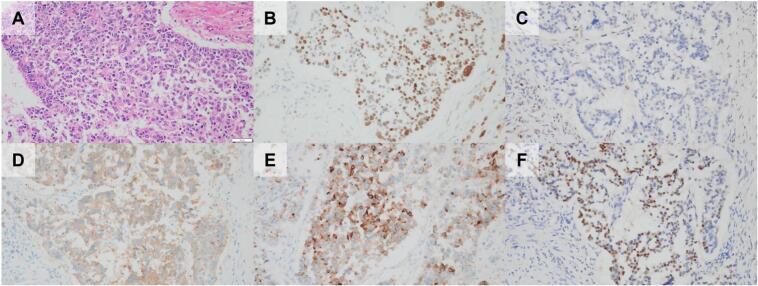
Microscopic images of the neuroendocrine carcinoma component/component Z. A, hematoxylin-eosin staining. The component was composed of solid nested growth of atypical cells with granular cytoplasm and dense chromatic nuclei. Scale bar = 50 μm. Immunohistochemically, the component showed p53 overexpression (B), loss of Rb expression (C), expression of synaptophysin (D), chromogranin A (E), and insulinoma-associated protein 1 (F).

Macroscopically, a 35 mm-sized, relatively well-demarcated, whitish solid mass was detected in the pancreatic head. Multicystic dilatation of the pancreatic ducts was also detected on the distal aspect of the pancreas (Fig. 1C–F). Microscopically, the mass comprised the following four components: W, X, Y, and Z, occupying 40 %, 5 %, 40 %, and 15 % of the tumor area, respectively. Component W was composed of atypical epithelial cells with eosinophilic cytoplasm, forming irregular trabecular or poorly defined glandular structures surrounded by desmoplastic stroma (Fig. 2A). Component X was composed of sheet-like growth of polygonal cells (Fig. 2D). Component Y was composed of tubulopapillary growth of tall columnar cells with clear-to-granular cytoplasm and centrally located nuclei. The fibrotic stroma was less than that of Component W (Fig. 3A). Component Z was composed of a solid nested growth of intermediate-sized polygonal cells with granular cytoplasm and relatively small but dense chromatic nuclei (Fig. 4A).

Immunohistochemically, Component X was focally (<50 % of the component) positive for p40, a squamous cell marker (Fig. 2E); Y was diffusely positive (≥50 % of the component) for glypican-3 (GPC3) and spalt-like transcription factor 4 (SALL4), and focally positive for alpha-fetoprotein (AFP) (Fig. 3D–F); Z was diffusely positive for neuroendocrine markers such as synaptophysin, chromogranin A, and insulinoma-associated protein 1 (INSM1) (Fig. 4D–F); however, W was negative for p40, AFP, GPC3, SALL4, synaptophysin, chromogranin A, and INSM1. Interestingly, p53 overexpression and loss of Rb expression were observed in all components (Figs. 2BC, 3BC, and 4BC). Cytokeratin (CK) AE1/AE3, CK7, and mucin (MUC) 1 were positive in all components, whereas CK20, MUC5AC, and MUC6 showed focal expression, and MUC2, arginase-1, hepatocyte paraffin 1, caudal-type homeobox 2, trypsin, and B-cell lymphoma 10 were negative for all components. Ki-67 labelling index in components W, X, Y, and Z were 40 %, 50 %, 80 %, and 60 %, respectively. A summary of the immunohistochemical results is shown in Table 1.

**Table 1:** Summary of immunohistochemical findings.

	PDAC	SCC	AED	NEC
CK AE1/AE3	+	+	+	+
CK7	+	+	F	+
CK20	F	−	−	−
MUC1	+	+	+	F
MUC5AC	−	−	F	−
MUC6	F	−	F	−
p40	−	F	−	−
AFP	−	−	F	−
GPC3	−	−	+	−
SALL4	−	−	+	−
Syn	−	−	−	+
CgA	−	−	−	+
INSM1	−	−	−	+
p53	OE	OE	OE	OE
Rb	L	L	L	L
Ki-67 labeling index	40 %	50 %	80 %	60 %

PDAC, pancreatic ductal adenocarcinoma; AED, adenocarcinoma with enteroblastic differentiation; NEC, neuroendocrine differentiation; CK, cytokeratin; MUC, mucin; AFP, alpha-fetoprotein; GPC3, glypican-3; SALL4, spalt-like transcription factor 4; Syn, synaptophysin; CgA, chromogranin A; INSM1, insulinoma-associated protein 1; +, diffusely positive (≥50 % of each component stained); F, focally positive (<50 % of each component stained); −, negative; OE, overexpression; L, Loss of expression. MUC2, arginase-1, hepatocyte paraffin 1, caudal-type homeobox 2, trypsin, and B-cell lymphoma 10 were negative for all components.

Thus, we considered Component W as conventional PDAC, X as squamous cell carcinoma (SCC), Y as AED, and Z as NEC. Thus, we diagnosed the tumor as a PDAC with enteroblastic, neuroendocrine, and squamous differentiation. The PDAC and SCC components invaded the duodenal wall, and the PDAC component also invaded extrapancreatic tissue. Lymphovascular and perineural invasion was present; however, lymph node metastasis was absent. The stage of tumor was classified as pT2N0M0, stage IB in 8th edition of TNM classification by Union for International Cancer Control. The surgical margins were negative.

The multicystic lesion was covered by gastric foveolar-type epithelium with mild-to-focally moderate atypia forming finger-like papillae; thus, we diagnosed it as a gastric-type low-grade intraductal papillary mucinous neoplasm (IPMN, Fig. 2D). The IPMN involved both the branch and main pancreatic ducts, but was located 8 mm away from the carcinoma. Moreover, aberrant p53 expression or loss of Rb expression was not observed in the IPMN.

Adjuvant chemotherapy with tegafur, gimeracil, and oteracil potassium was initiated three months postoperatively. However, multiple liver metastases were found five months postoperatively. Subsequently, he received second- and third-line chemotherapy; however, he died of bath-related cardiac arrest 14 months postoperatively.

## 3 Discussion

AED is a carcinoma with tubulopapillary growth of columnar cells with clear cytoplasm expressing oncofetal proteins, including AFP, GPC3, and/or SALL4 [[1], [2], [3]]. In the current WHO classification of tumors, gastric AED (GAED) is newly listed as a related entity of hepatoid adenocarcinoma (HAC), a primary extrahepatic carcinoma composed of eosinophilic hepatocyte-like cells showing a solid sheet-like growth pattern. Because GAED and gastric HAC (GHAC) often coexist or overlap, and share similar clinicopathological and genetic features, such as aggressive clinical course, frequent lymphovascular invasion, lymph node and liver metastases, admixture with conventional adenocarcinoma components, and *TP53* mutation and *ERBB2* amplification [[1], [2], [3]]. However, GAED should be differentiated from GHAC because it has a different morphology and better prognosis [5].

>50 % of HAC/AED cases occur in the stomach, but rarely in other organs including the pancreas [[6], [7], [8], [9], [10]]. However, the frequency of extragastric HAC (EGHAC) is much lower than that of GHAC. Moreover, extragastric AED (EGAED) are much rarer than EGHAC and have not yet been listed in the WHO classification. We searched the PubMed database and found a few case reports or series of esophageal, colorectal, and biliary AED [[11], [12], [13], [14], [15]]; and only one case report of pancreatic AED that was written in Chinese [16]. However, there are tumors showing AED morphology in a case series of “hepatoid” tumor of the bile duct [8]. EGAED seems to be underrecognized or confused with HAC owing to its novelty.

The clinicopathological features of EGHAC/AED remain controversial. For example, colorectal AED appear to show frequent lymphovascular invasion, lymph node metastasis, and distant metastases [2]. In contrast, pancreatobiliary HAC/AED vary in the degree of lymphovascular invasion, lymph node metastasis, and clinical outcome [7,8,15]. This controversy may be caused by under-recognition or confusion of entities. Due to its rarity, the molecular features of EGHAC/AED remain unclear. *TP53* was reported as the only commonly mutated gene occurring in hepatoid tumors across different sites; however, the number of cases in each organ (colon 2/3, esophagogastric 2/4, biliary 2/4, genital 1/6, and lung 1/2 cases) was too few to make firm conclusions [17].

In our case, AED was mixed with conventional PDAC and NEC. Admixtures of both conventional adenocarcinoma and NEC have occasionally been reported in EGAED and pancreatic HAC [7,11,12,14]. As mentioned above, a mixed conventional adenocarcinoma component, suggesting that HAC/AED is transformed from conventional adenocarcinoma, is common in GHAC/AED. In contrast, mixed GHAC-NEC is extremely rare, with only seven reported cases [18], and there have been no reports of mixed GAED-NEC. Mixing with NEC appears to be a characteristic phenomenon in EGHAC/AED cases.

NEC, an aggressive and poorly differentiated carcinoma showing neuroendocrine differentiation, is rare but can occur in various organs including the pancreas [1]. Some NEC is mixed with a variable amount of non-neuroendocrine carcinoma, such as adenocarcinoma, suggesting that NEC is also transformed from non-neuroendocrine carcinoma [1,19]. NEC of various organs, including the pancreas, is characterized by frequent *TP53* and *RB1* mutations [1,19]. However, the significance of *TP53* mutations is limited, because they are frequently observed in PDAC [20]. In contrast, *RB1* mutations are rare in conventional PDAC [20] and can be key molecules in revealing the histogenesis of mixed NEC cases.

In our case, both p53 overexpression and Rb loss were observed in all the components, indicating *TP53* and *RB1* mutations, respectively. These genetic findings suggest that non-neuroendocrine components have the potential to transform into NEC. In other mixed adenocarcinoma-AED-NEC cases, p53 overexpression in all components has also been frequently observed [11,13,14]; however, Rb status has not been well investigated, except in an esophageal case showing Rb loss only in the NEC component [14]. These findings differ from the findings in our case and suggest that Rb was lost during the transformation from adenocarcinoma or AED to NEC. There may be several different carcinogenetic pathways for mixed adenocarcinoma-AED-NEC, which is consistent with the findings of previous studies on gastroenteropancreatic NEC [19].

## 4 Conclusion

AED is a rare histological subtype related to HAC and can occur in various organs. GAED is relatively well accepted, and its features are relatively well understood. In contrast, only a few EGAED cases, particularly in the pancreas, have been reported to date. The features of EGAED remain unclear because of its rarity, under-recognition, or confusion with EGHAC. More EGAED case reports or series are required to establish EGAED.

EGAED combined with NEC is complex but has occasionally been reported. The status of Rb expression or *RB1* alteration, which is a characteristic of NEC, may be the key to resolving the histogenesis of mixed AED-NEC cases.

## Ethical approval

Our institution (Kobe University Hospital) does not require ethics committee approval for Case Reports. For human subjects, the investigation was conducted in accordance with the Declaration of Helsinki of 1975.

## Funding

This research did not receive any specific grant from funding agencies in the public, commercial, or not-for-profit sectors.

## Author contribution

Takayuki Kodama: Conceptualization, Investigation, Formal Analyses, Writing–Original Draft.

Akiho Tani: Resources, Investigation.

Hisoka Yamane: Resources, Writing–Review and Editing.

Tomoo Itoh: Investigation, Supervision.

All authors read and approved the final manuscript.

## Guarantor

Takayuki Kodama accepts all responsibility of this article.

## Research registration number

1. Name of the registry: UMIN Clinical Trials Registry (UMIN-CTR)

2. Unique identifying number or registration ID: UMIN000054196

## Conflict of interest statement

All authors declare that they have no conflicts of interest.
